# Cyanoacrylate injection treatment for postoperative leakage of Boerhaave's syndrome: A case report

**DOI:** 10.1097/MD.0000000000028075

**Published:** 2021-12-10

**Authors:** Su Bee Park, Yun Jin Yum, Jae Myung Cha

**Affiliations:** Department of Internal Medicine, Kyung Hee University Hospital at Gang Dong, Kyung Hee University School of Medicine, Seoul, Korea.

**Keywords:** anastomotic leakage, Boerhaave's syndrome, case report, cyanoacrylate, endoscopic intervention

## Abstract

**Rationale:**

Surgical treatment remains the most effective option for treating Boerhaave's syndrome. However, in cases of postoperative anastomotic leakage of Boerhaave's syndrome, endoscopic interventions such as over-the-scope clip, stenting, or cyanoacrylate injection have emerged over reoperation.

**Patient concerns:**

We report the case of a 50-year-old male patient who presented with vomiting and abdominal pain after alcohol consumption. Laparoscopic surgery was performed for primary closure of a laceration at the lower esophagus, and for the closure of a Boerhaave's syndrome, which was detected by abdominal computed tomography. However, postoperative anastomotic leakage was confirmed through esophagography after the operation. In our case, endoscopic treatment with an over-the-scope clip and stenting were not effective for the repair of the anastomotic leakage, but cyanoacrylate injection successfully healed the anastomotic leakage.

**Diagnoses:**

Boerhaave's syndrome was initially detected by abdominal computed tomography, but postoperative anastomotic leakage after the operation was confirmed with esophagography.

**Interventions:**

A total of 2.0 cc of N-butyl-2-cyanoacrylate and lipiodol mixture (at 1:1) was injected into the leakage tract through the perforation entrance.

**Outcomes:**

Complete healing of the anastomotic leakage was confirmed with a follow-up esophagoscopy.

**Lessons:**

N-butyl-2-cyanocrylate injection treatment can be used as a rescue option for postoperative leakage when over-the-scope clips and stenting fail for this indication.

## 1 Introduction

Boerhaave's syndrome is a spontaneous rupture of the esophagus attributed to sudden increase in intraesophageal pressure, and its mortality is higher than that of other types of esophageal ruptures.^[1]^ Usually, surgery and conservative management are the major treatment options for Boerhaave's syndrome; however, postoperative anastomotic leakage is often associated with morbidity and mortality. Postoperative anastomotic leakage may be effectively managed with endoscopic procedures,^[2]^ such as endoscopic clipping, stenting, and cyanoacrylate injection,^[2]^ each of which has its own advantages and disadvantages. Cyanoacrylate injection is a well-recognized method for the treatment of gastrointestinal fistulas.^[3]^ However, cyanoacrylate injection treatment can be used as a rescue option for postoperative leakage when over-the-scope clip (OTSC) and stenting fail to heal the postoperative leakage.^[3–5]^

We report a case of postoperative anastomotic leakage of a Boerhaave's syndrome, which was completely healed with cyanoacrylate injection.

## 2 Case presentation

**Figure 1:**
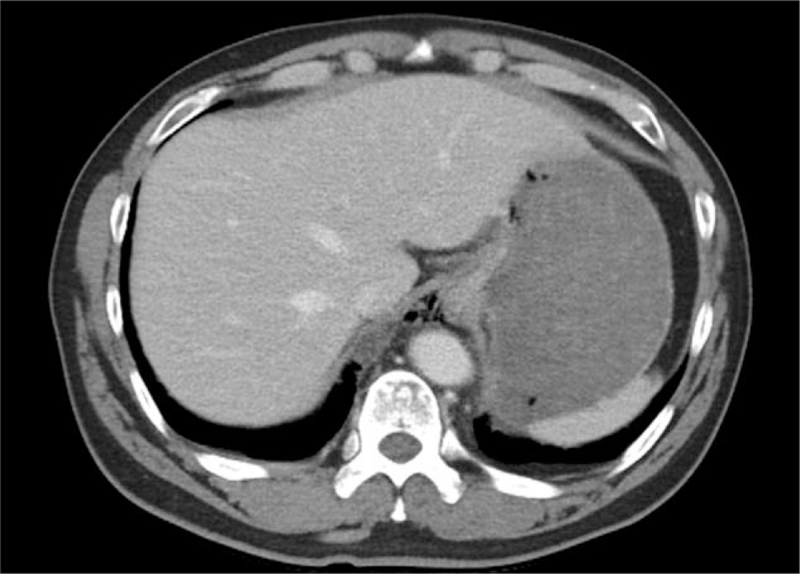
Computed tomography of the abdomen showing extraluminal air bubbles at the paraesophageal space, around the gastroesophageal junction, and the lesser sac. Focal wall defect of the lower esophagus suggestive of esophageal perforation.

**Figure 2:**
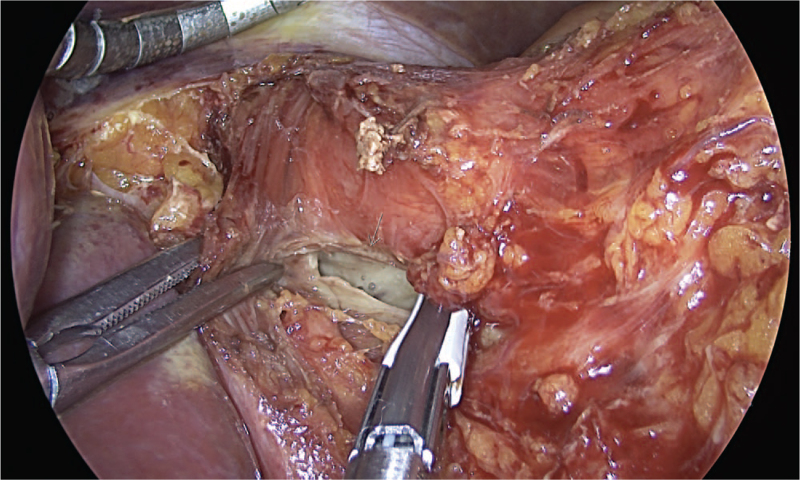
Laparoscopic view showing an approximately 5 cm laceration (white arrow) at the esophagogastric junction without adjacent tissue inflammation.

A 50-year-old man visited the emergency department with persistent right upper quadrant pain and vomiting. He was a chronic alcoholic who drank 1 or 2 bottles of Soju daily and often vomited after drinking. Before presentation, he had an episode of vomiting while brushing his teeth, with associated severe abdominal pain with a numerical rating scale of six points. Tenderness and rigidity were noted on physical examination of the upper abdomen. He had a medical history of hypertension and dyslipidemia. At the time of hospital visit, his vital signs were stable with a blood pressure of 150/100 mm Hg, pulse rate of 95/min, respiration rate of 18/min, and body temperature of 36.6°C. No significant findings were noted in the initial blood tests, including complete blood count, blood chemistry, cardiac markers, and C-reactive protein. Abdominal computed tomography (CT) showed extraluminal air bubbles around the paraesophageal area, esophagogastric junction, and a focal wall defect of the lower esophagus (Fig. 1). On the basis of the clinical history and CT findings, Boerhaave's syndrome was suspected. After the immediate commencement of systemic intravenous antibiotics, laparoscopic surgery was performed for the esophageal perforation. On the surgical field, an approximately 5 cm sized laceration, extending through one-third of the wall's thickness, was noted at the gastroesophageal junction (Fig. 2), and primary closure was performed using the monifix and omental patch. After the operation, he was fasted for 5 days and given parenteral nutrition, which relieved his pain.

**Figure 3:**
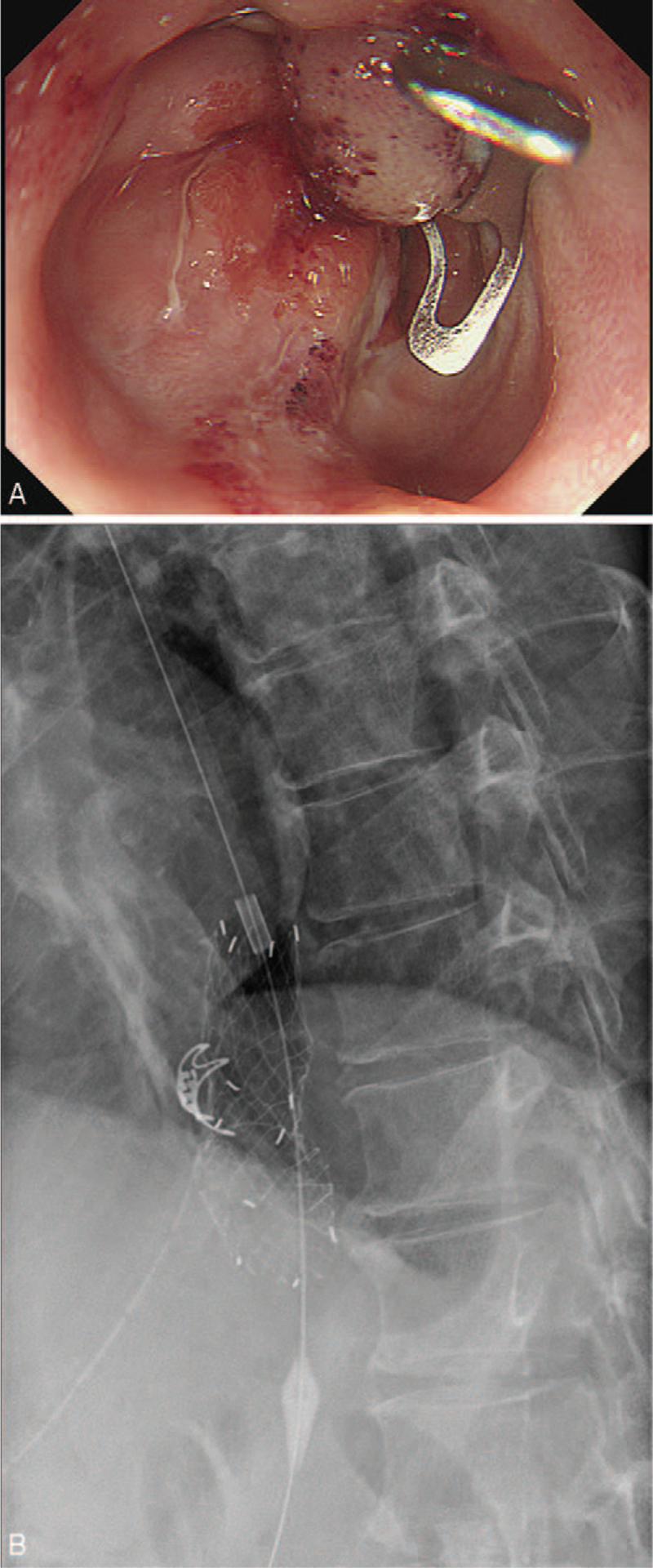
For the primary closure of the anastomotic leakage, over-the-scope clip was attempted for the 15 mm perforation, but this was unsuccessful due to the presence of fibrotic tissue at the perforation site, which prevented efficient suctioning (A). As a second option, a 6 cm anti-migration covered metal stent was placed at the esophagogastric junction (B).

On the fifth day postoperation, esophagography showed extraluminal contrast leakage at the anastomotic site. Esophagoscopy showed an approximately 15 mm perforation at the site of the anastomosis at the esophagogastric junction. Endoscopic closure with OTSC was initially performed for the primary closure (Fig. 3A); however, effective closure was difficult due to scarring and presence of fibrotic tissue around the perforation site, which prevented efficient suctioning of the surrounding area. An anti-migration covered metal stent was planned as a secondary treatment, as he had a high fever of 39°C, and a follow-up esophagography showed persistent extraluminal contrast leakage at the anastomotic site. A 6 cm anti-migration covered metal stent (Hanaro Shim's stent, M.I.Tech Co., Ltd., Gyeonggi-do, Korea) was successfully placed at the gastroesophageal junction (Fig. 3B). To prevent stent migration, a nylon thread, which was connected to the proximal part of the stent, was hung around the patient's ear. Despite efforts to prevent stent migration, the stent dislodged into the stomach five days after the procedure. Fortunately, extraluminal contrast leakage was not observed on follow-up esophagography, and extraluminal air bubbles around the site of the anastomosis had decreased on abdominal CT. There were no specific findings in his clinical symptoms and laboratory tests; therefore, he was discharged with an assessment of improvement of postoperative anastomotic leakage after stenting.

**Figure 4:**
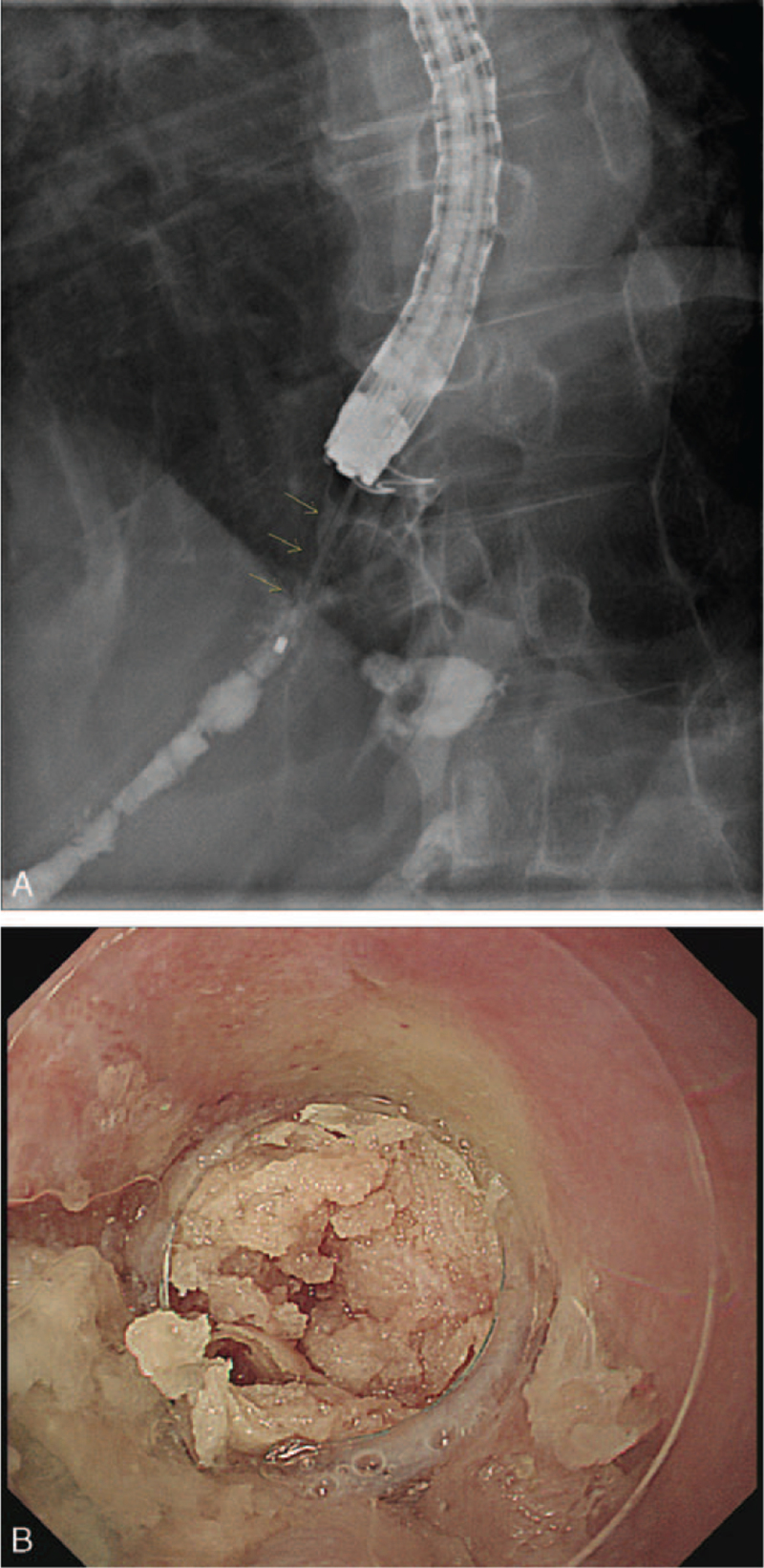
On fistulograms, an approximately 2.5 cm leakage tract (yellow arrow) was noted when contrast dye was injected into the perforation entrance (A). A volume of 2.0 cc of N-butyl-2-cyanoacrylate and lipiodol mixture at 1:1 was injected into the leakage tract (B).

**Figure 5:**
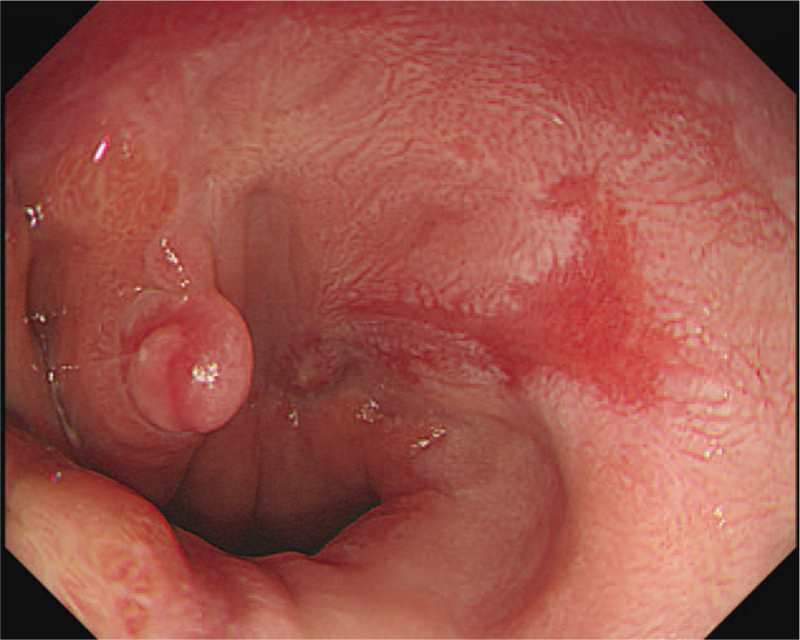
Follow-up esophagoscopy showing complete healing of the previous postoperative leakage.

However, 9 days after discharge, he was re-hospitalized on account of abdominal pain and fever. Blood tests showed increased inflammatory markers with a C-reactive protein of 4.9 mg/dL (0–0.5, normal range). Esophagoscopy showed a perforation at the anastomosis site, and an approximately 2.5 cm slender leakage tract was traced when a contrast dye was injected into the perforation site (Fig. 4A). As a rescue treatment, 2.0 cc of N-butyl-2-cyanoacrylate (Histoacryl, B. Braum Korea Co., Ltd., Seoul, Korea) and lipiodol mixture (at 1:1) was injected into the leakage tract through the perforation (Fig. 4B). He showed improvement in the abdominal pain and on laboratory findings a day after the procedure. On the second day after operation, he started a clear liquid diet and was fine with a full liquid diet on the third day. Finally, the patient was discharged on solid diet and oral antibiotics. A month after cyanoacrylate treatment, a complete scar formation was observed at the previous perforation site, and the previous anastomotic leakage site had healed completely (Fig. 5). Up to now, the patient has been followed up at the outpatient clinic for eight months without recurrence.

## 3 Discussion

Boerhaave's syndrome is an effort rupture of the esophagus that occurs when the cricopharyngeal muscle fails to relax properly in a situation in which esophageal and thoracic pressures rise due to straining or vomiting.^[6,7]^ Clinicians may suspect this diagnosis when patients experience severe pain in the neck, chest, or upper abdomen after vomiting. However, it is difficult to diagnose this disease with symptoms alone because nonspecific symptoms are often presented. Currently, surgery remains the most effective treatment option for Boerhaave's syndrome,^[1]^ as we did in our case. However, postoperative anastomotic leakage may occur, which is often associated with morbidity and mortality. Recently, postoperative anastomotic leakage has been effectively managed with endoscopic procedures,^[2]^ such as OTSC, stenting, and cyanoacrylate injection. In our case, OTSC and covered metal stenting did not effectively heal the anastomotic leakage; however, cyanoacrylate injection was successful as a rescue therapy. Each endoscopic intervention has its advantages and disadvantages depending on the size and site of the leakage, the interval from operation to diagnosis, and the degree of damage to adjacent organs.

Cyanoacrylate injection is a well-recognized treatment method for gastrointestinal fistula, and it can be used successfully for treating postoperative anastomotic leakage.^[3]^ Cyanoacrylate injection is indicated for small perforations, sized less than 10 mm.^[3,4]^ However, the perforation size was larger than that of the classic indication in our case; therefore, cyanoacrylate injection was not selected as the first treatment option. First, OTSC was attempted because it can effectively cover up to a maximum 2 cm sized perforation and ligate the full thickness of the layer.^[5]^ The expected effect was not obtained because the surrounding tissue of the perforation site was fibrotic, which prevented effective grasping of the perforation site. As a second option, a covered metal stent was selected, as natural healing can be expected when the perforation did not last for a long time or there was no maturation tract.^[4]^ Unfortunately, migration may prevent the healing of the anastomotic leakage, as in our case.

Cyanoacrylate was first synthesized in 1949 by Ardis as a tissue adhesive that polymerizes quickly on contact with tissue fluid. Since then, derivatives that are less harmful to the human body have been produced; after the 1970 s, N-butyl-2-cyanocrylate (Histoacryl), which is known to have the least tissue inflammatory action, has been used most commonly.^[8]^ In the 1980 s, it was first introduced for endoscopic hemostasis of variceal bleeding. The effect and safety of N-butyl-2-cyanocrylate in gastrointestinal fistula has also been proven in several reports.^[9]^ As a method of injecting N-butyl-2-cyanocrylate into the fistula, both endoscopic and radiological intervention are possible. A proper indication for endoscopic N-butyl-2-cyanocrylate infusion is a fistula opening measuring approximately 5 to 7 mm, but it is difficult to consider N-butyl-2-cyanocrylate injection when the fistula opening is larger than 1 cm. In addition, N-butyl-2-cyanocrylate injection is unsuitable in patients with drainage through the fistula of more than 200 mL per day, stenosis distal to the fistula, inflammatory or abscess-formed fistula, or fistulas arising from Crohn's disease or malignant tumors.^[5]^ In a Japanese phase II study, 22 (88%) of 25 patients experienced complete closure of their esophageal fistulas without complications after endoscopic injection of the alpha-cyanoacrylate monomer.^[9]^ Our case highlights the role of N-butyl-2-cyanocrylate injection as a rescue therapy for postoperative leakage even when OTSC and stenting fail.

The patient has provided informed consent for publication of the case. The head of the gatroenterology department of Kyung Hee University Hospital at Gang Dong is in charge of the anonymization of the patient. Ethical approval was given by the ethics committee of Kyung Hee University Hospital at Gang Dong.

## Author contributions

**Conceptualization:** Jae Myung Cha.

**Resources:** Su Bee Park, Yun Jin Yum.

**Supervision:** Jae Myung Cha.

**Writing – original draft:** Su Bee Park.

**Writing – review & editing:** Su Bee Park, Jae Myung Cha.
